# Monitoring Sea Level in the Coastal Zone with Satellite Altimetry and Tide Gauges

**DOI:** 10.1007/s10712-016-9392-0

**Published:** 2016-11-18

**Authors:** Paolo Cipollini, Francisco M. Calafat, Svetlana Jevrejeva, Angelique Melet, Pierre Prandi

**Affiliations:** 1 National Oceanography Centre, European Way, Southampton, SO14 3ZH UK; 2 National Oceanography Centre, Brownlow Street, Liverpool, L5 3DA UK; 3 LEGOS, Université de Toulouse, CNES, CNRS, IRD, UPS, Toulouse, France; 4 Mercator Océan, 8-10 rue Hermès, 31520 Ramonville Saint-Agne, France; 5 Space Oceanography Division, CLS, 8-10 rue Hermès, 31520 Ramonville Saint-Agne, France

**Keywords:** Sea level, Coastal zone, Radar altimetry, Coastal altimetry, Tide gauge

## Abstract

We examine the issue of sustained measurements of sea level in the coastal zone, first by summarizing the long-term observations from tide gauges, then showing how those are now complemented by improved satellite altimetry products in the coastal ocean. We present some of the progresses in coastal altimetry, both from dedicated reprocessing of the radar waveforms and from the development of improved corrections for the atmospheric effects. This trend towards better altimetric data at the coast comes also from technological innovations such as Ka-band altimetry and SAR altimetry, and we discuss the advantages deriving from the AltiKa Ka-band altimeter and the SIRAL altimeter on CryoSat-2 that can be operated in SAR mode. A case study along the UK coast demonstrates the good agreement between coastal altimetry and tide gauge observations, with root mean square differences as low as 4 cm at many stations, allowing the characterization of the annual cycle of sea level along the UK coasts. Finally, we examine the evolution of the sea level trend from the open to the coastal ocean along the western coast of Africa, comparing standard and coastally improved products. Different products give different sea level trend profiles, so the recommendation is that additional efforts are needed to study sea level trends in the coastal zone from past and present satellite altimeters. Further improvements are expected from more refined processing and screening of data, but in particular from the constant improvements in the geophysical corrections.

## Introduction

In the previous paper in this issue, Ablain et al. explain in detail the importance of sea level rise, a measure of the increase in ocean volume, as a clear indicator of climate change and one of its main effects. Satellite altimeter-derived sea level rise is now well quantified both as a global mean and in its geographical distribution (as, respectively, visible in Figs. 2, 5 of Ablain et al., 2016) thanks to multiple efforts that include the sea level climate change initiative (Ablain et al. 2015). Satellite-based measurements of sea level compare well with the measurements from the global tide gauge network (Fig. 8 of Ablain et al., 2016). The requirement prescribed by the Global Climate Observing System (GCOS) of an accuracy better than 0.3 mm/year in the altimeter-derived rate of global mean sea level rise is still not fully met; however, the improvements seen in the last few years make that target a realistic one. The future looks promising for the precise determination of global and regional sea level rise from the integration of altimetry and tide gauges.

However, there is still an observational gap in our knowledge, and this gap is in the region that represents the main interface between our society and the ocean, i.e. the coastal zone. While tide gauges are usually located at the coast therefore providing coastal sea level measurements (those are relative to a local datum as discussed in Sect. 6 of Ablain et al., 2016, but can be made absolute in the presence of accurate GPS positioning), altimeters have difficulties in the coastal zone. Altimeter data are normally flagged as bad and discarded shorewards of 10–50 km from the coast depending on the particular instrument and the local coastal morphology. As a consequence, all studies combining or contrasting altimeter-derived and tide gauge-derived sea level rise have been essentially comparisons between rates in different locations, and to our knowledge no study has filled this observational gap as yet. Filling the gap, which may at first sound like a purely academic exercise, becomes important when the following two factors are considered:

1. All impacts of sea level rise on society and ecosystems are going to be suffered entirely at the coast. As an example, a recent study (Hauer et al. 2016) found that a rise of only 90 cm by 2100 would put 4.2 million people at risk of inundation in the coastal zone for the continental USA alone. When projected globally and considering highly vulnerable areas such as low-lying island and deltas, the number of people that would be flooded if not relocated quickly rises to reach the order of 100 million or more (Hinkel et al. 2014).
2. Many stretches of the world’s coast still do not possess in situ sea level measuring devices, and those stretches include many vulnerable regions in developing countries. Altimetry is at present the only way of obtaining measurements of sea level variations in those regions and can already offer 24 years of observations from the TOPEX/Jason-1/2/3 ‘reference’ series and from the ERS–Envisat–AltiKa series,^1^ so it will remain valuable in order to extend the sea level record back in time also when tide gauges will eventually start to be installed in those regions.

It is therefore of great importance to be able to link the satellite altimeter measurements of sea level rise with the tide gauge measurements, by bridging the open-ocean measurements with those in close proximity to the coast. This has been one of the motivations that since the early 2000s have led to the development of the field of coastal altimetry (Vignudelli et al. 2011), which aims at recovering more numerous and better measurements of sea surface height in the coastal strip. Processing enhancements and improvements in the corrections is at the basis of a number of improved coastal altimetry datasets now becoming available, than can potentially be used for sea level studies but will need a thorough validation for that purpose.

In this contribution, we first review the status of the measurements of sea level in the coastal zone from tide gauges (Sect. 2), and then in Sect. 3 we examine the same issue from the point of view of coastal altimetry, with an overview of the improvements in the retrieval of sea surface height from altimetry in the coastal zone which have been made possible by algorithmic improvements and development of better corrections and data editing. This section also presents the datasets available for altimetry and coastal altimetry, for the benefit of the potential users of those products. Some particularly promising prospects for the monitoring of sea level in the coastal zone come from the advent of new technologies, whose coastal performance is discussed in Sect. 4: these technologies are Ka-band altimetry from AltiKa, and SAR altimetry from CryoSat-2 (and now Sentinel-3). We then present two specific examples of coastal sea level monitoring: in Sect. 5, a case study showing how local sea level can be monitored with altimetry and tide gauges around the coast of the UK and, in Sect. 6, some results on the variations of sea level rise rate as a function of distance from shore along the West African coast. The concluding section translates the science reviewed and results presented into recommendations for refinements to the processing, which should lead to further progress of the field.

## Monitoring Sea Level with Tide Gauges

Tide gauges have been used since ancient times to measure sea level changes at the coast. In Amsterdam, an historical record of observations of sea level changes using a pole provides evidence of sea level rise and variability since 1700 (Van Veen 1945). Several tide gauge records from locations in Europe, for instance Liverpool since 1768 (Woodworth 1999) and Stockholm since 1774 (Ekman 1988) greatly contribute to our understanding of sea level changes over the eighteenth century. Since the 1830s, automatic or self-registering tide gauges were developed. The first automatic tide gauges are often credited to those installed in the Thames Estuary, England (Matthäus 1972). The first automatic tide gauge outside Europe was installed in San Francisco, USA in 1851. By the end of the nineteenth century, automatic tide gauges had been installed at many ports (Baltic and Mediterranean Seas, the USA coast). A network global in scope was starting to appear. Sea level rise during the twentieth century is estimated using almost 1000 tide gauge locations (e.g., Douglas 1997; Jevrejeva et al. 2006, 2014; Church and White 2006, 2011; Hay et al. 2015), with techniques accounting for the fact that most of those tide gauges do not cover the entire twentieth century.

**Fig. 1:**
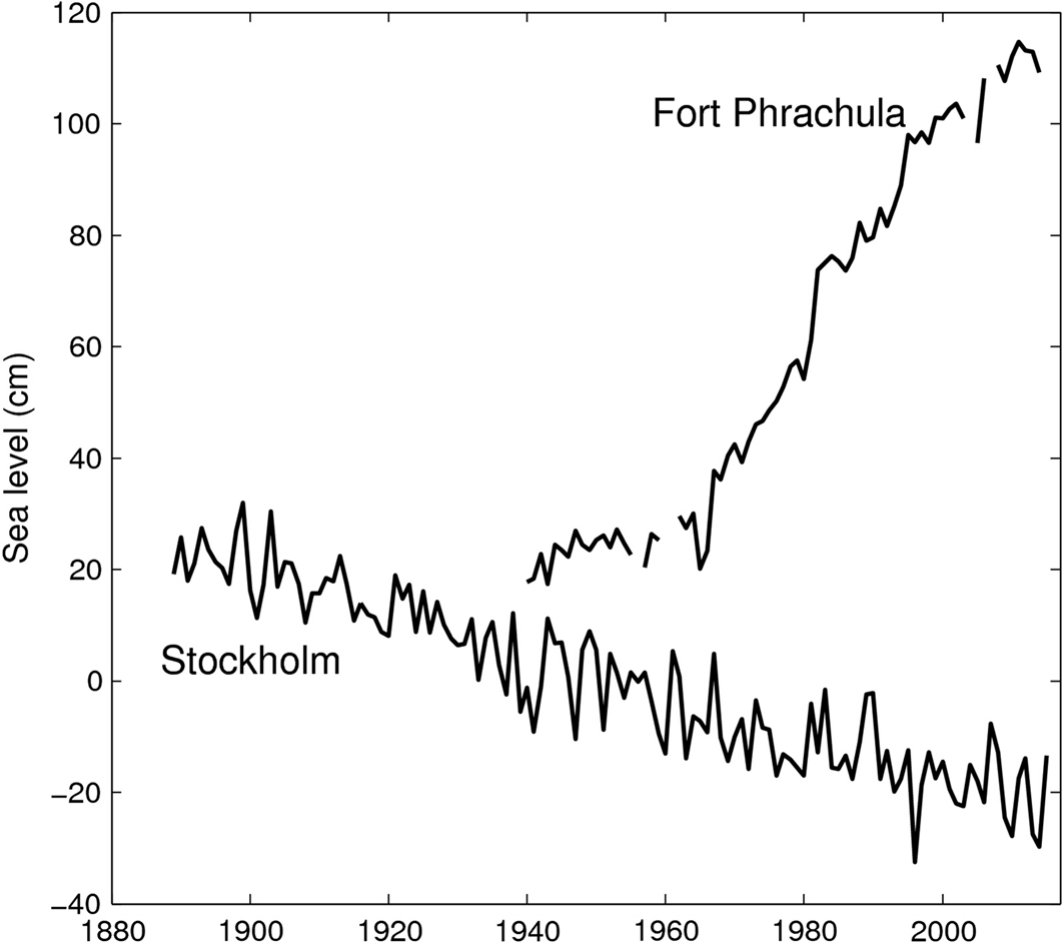
Annual values of sea level from the tide gauge records at Fort Phrachula (Bangkok, Thailand) and Stockholm (Sweden)

Tide gauge data provide valuable information about sea level changes from a few seconds to centuries at the locations where they are installed; however, these observations suffer from several limitations:

- the geographical distribution of tide gauges is naturally confined to the continental margins and some ocean islands, which provides poor sampling of the ocean basins; in addition, most tide gauges are located in the Northern Hemisphere (Europe, Japan and the USA);
- available tide gauge records do not all cover the same time period, and their number decreases rapidly as we go back in time, especially prior to 1960;
- tide gauges are attached to the land, providing measurements relative to the Earth’s crust, which could move. Vertical land movement is the one of the main difficulties to interpret tide gauge measurements (Wöppelmann and Marcos 2016; Jevrejeva et al. 2014). Some extreme examples (Fig. 1) of the effect that vertical land motion can have on local sea level are Fort Phrachula, near Bangkok, where relative sea level has been rising at a rate of about 15 mm/year since the 1960s due to subsidence caused by increased groundwater extraction, or Stockholm where it is falling at a rate of 3.8 mm/year due to crustal uplift associated with glacial isostatic adjustment.
- there is no common reference level for the individual tide gauge records, despite some clear recommendations for it (for instance Woodworth et al. 2013), and this creates a problem of stacking records together.

It should be noted that for long-term (climate) applications the temporal variation of the tidal constituents must also be understood and accounted for; these are best studied in detail with a high-frequency (i.e. hourly or more frequent) tidal datasets such as the Global Extreme Sea Level Analysis (GESLA) dataset and its follow-on GESLA-2: this issue is examined in detail in Woodworth (2010).

**Fig. 2:**
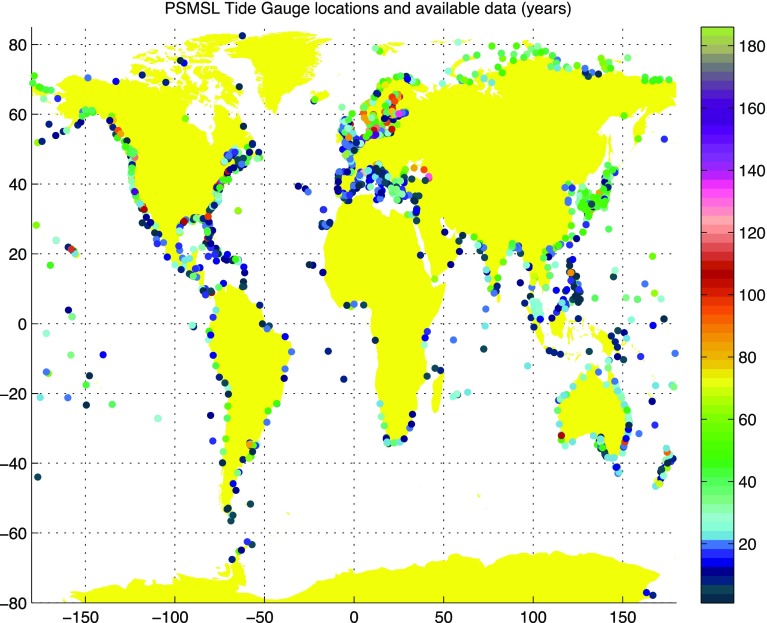
Locations of tide gauges in the PSMSL database for which annual mean sea level information is available and the number of available annual records in each station

Since 1933, tide gauge records have been collected and distributed by the Permanent Service for Mean Sea Level (PSMSL). There are records from more than 2000 locations (Fig. 2) available from PSMSL via the webpage www.psmsl.org (Holgate et al. 2012). An international programme, the Global Sea Level Observing System (GLOSS), is established under the Joint Technical Commission for Oceanography and Marine Meteorology (JCOMM) of the World Meteorological Organization (WMO) and the Intergovernmental Oceanographic Commission (IOC), with the goal of creating high-quality global and regional sea level networks for application to climate, oceanographic and coastal sea level research. There are 289 sea level stations (global core network) around the world designed to provide an approximately evenly distributed sampling of global coastal sea level variations. GLOSS sites, which include Global Positioning System (GPS) receivers to monitor vertical land movements, contribute to long-term climate change studies such as those of the WMO-UNEP Intergovernmental Panel on Climate Change (IPCC).

**Fig. 3:**
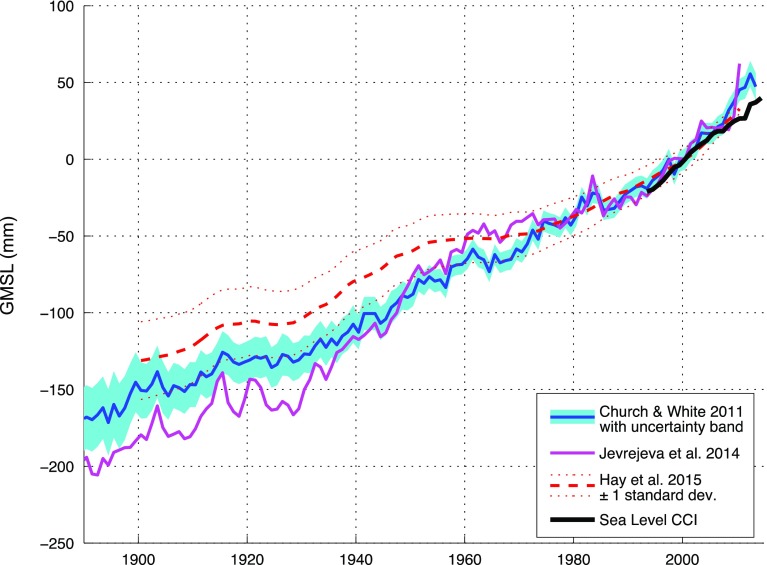
Global mean sea level (GMSL) from various reconstructions cited in the text and altimetry

Tide gauge observations are used to measure and predict tides, quantify the size of tsunamis and storm surges. Tide gauge records are widely used in coastal engineering for design of coastal infrastructure. Sea level datasets from tide gauges are utilized in many scientific disciplines, e.g., geodesy, oceanography, geology, paleo-oceanography studies and climatology. Selected tide gauges, in particular those located on islands, are also used for altimeter calibration. The most familiar application of tide gauge data is global and regional sea level rise and variability, providing information on long-term changes in global sea level during the last two centuries. Individual tide gauge observations (Douglas 1997), global sea level reconstructions using tide gauge data (Gornitz et al. 1982; Jevrejeva et al. 2006, 2008, 2014; Grinsted et al. 2007; Merrifield et al. 2009; Ray and Douglas 2011) and reconstructions that jointly use satellite altimetry and tide gauge records (Church and White 2006, 2011) or apply Bayesian fingerprinting techniques to tide gauge observations (Hay et al. 2015) show the evolution of sea level rise for the past 50–100 years. Three of these reconstructions, i.e. Church and White (2011), Jevrejeva et al. (2014) and the ‘Kalman smoother’ reconstruction by Hay et al. (2015) are shown in Fig. 3 alongside the altimeter-derived global mean sea level from the ESA Sea Level Climate Change Initiative (CCI) (Ablain et al. 2015). This illustrates the encouraging agreement between in situ-based and satellite-based quantifications of the global mean sea level.

## Monitoring Sea Level with Coastal Satellite Altimetry

Satellite altimetry has been one of the workhorses of open-ocean operational oceanography and global sea level monitoring, so efforts are naturally being made to use it also in the coastal zone. The main motivation is its spatial and temporal coverage: altimetry is global in space, covering even the most remote areas of the oceans (and the polar oceans with some satellites), and we have already 24 years of data from missions with accuracies of the order of just a few cm, starting with the ERS-1 launch in 1991.^2^ Moreover, in addition to sea level, it provides measurements of significant wave height (SWH) and wind.

**Fig. 4:**
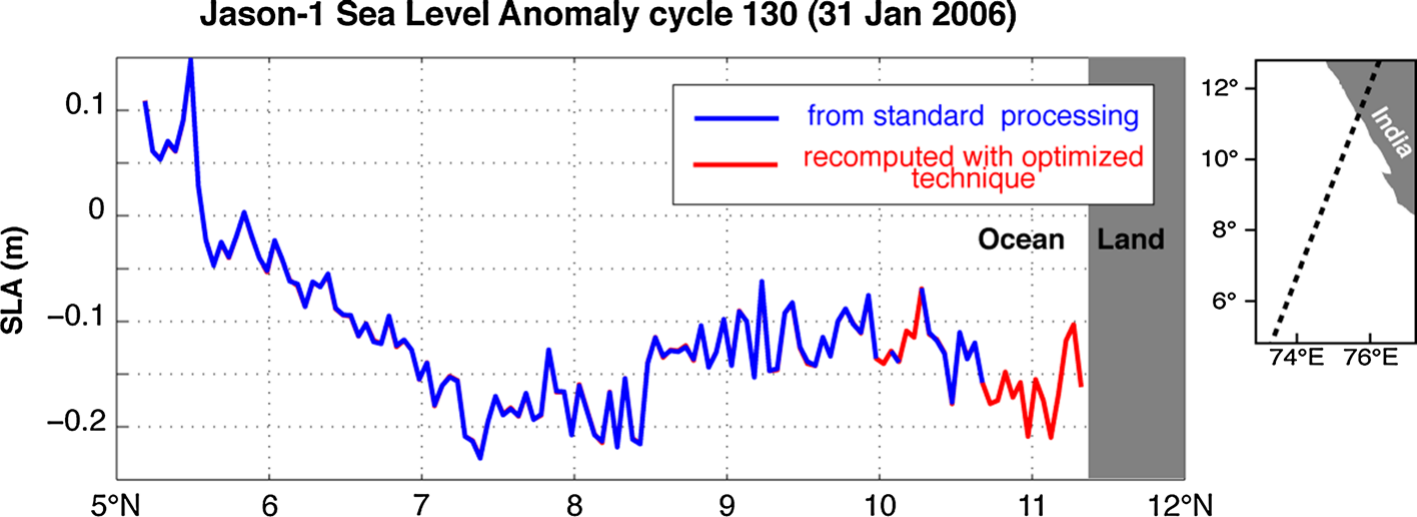
Example to illustrate the concept of coastal altimetry: profile of sea level anomaly along Jason-1 pass #003, cycle 130 crossing the south-west coast of India. In *blue,* the portion of the profile where all the data flags are set to ‘valid’ in the conventional data products. In *red,* the portion of the profile that can be recovered with optimized processing as described in the text

A simple illustration of the goal of coastal altimetry is in Fig. 4, showing the profile of sea level anomaly (SLA, i.e. the anomaly of the sea surface height w.r.t. its temporal mean) along Jason-1 pass #003, cycle 130 as it crossed the south-west coast of India on 31 January 2006. The portion of the profile where all the data flags are set to ‘valid’ in the conventional data products is plotted in blue and excludes several tens of km close to the coast, i.e. all the portion north of 10.7°N. Further gaps are seen in the data are between 10° and 10.2°N, possibly due to problems with some of the corrections or to problems with waveform retracking which could be due for instance to the presence of surface slicks: often, coastal altimetry techniques allow the recovery of meaningful measurements also in such problematic open-ocean circumstances. The portion in red is what can be recovered with an improved processing, which in this particular example consisted of de-flagging combined with customized screening of the corrections (i.e. using different validity ranges).

A lively international community of scientists has been involved in research and development of techniques for coastal altimetry in the last 10 years, with substantial support from space agencies such as the European Space Agency (ESA) and the Centre National d’Études Spatiales (CNES) as well as other research institutions, via several projects, amongst which:

- CNES’ PISTACH (2007–2011) for Jason-2 and ESA’s COASTALT (2008–2012) for Envisat, both developing specialized waveform retracking (see 3.1) and corrections.
- The X-TRACK initiative by the Centre of Topography of the Oceans and the Hydrosphere (CTOH) at LEGOS—Laboratoire d’Etudes en Géophysique et Océanographie Spatiales. X-TRACK products are derived for all precise altimeter missions by means of coastal-oriented screening of altimeter data and corrections (see Birol et al. 2016) and are used for the example on variability of sea level trends in the coastal zone in Sect. 6.
- The PEACHI products for AltiKa (Valladeau et al. 2015) currently produced by CLS (Collecte Localisation Satellites) under CNES funding. An assessment of these products is in Sect. 4.1.

Table 1 lists the available products for open-ocean and coastal and highlights those products that are provided at a higher post rate (20 or 40 Hz, corresponding to along-track distances of approximately 350 and 175 m, respectively) and are therefore more amenable to coastal altimetry applications. The coastal altimetry community (http://www.coastalt.eu/community) holds regular workshops where the science and techniques of coastal altimetry are reviewed and the various applications are showcased and discussed.

**Table 1:** Available products for open-ocean and coastal altimetry as of October 2016

ID	Produced by	Altimeter	Product level	Posting rate	Coverage	Download from	Comments
AVISO	CLS, CNES CNES	e1, tx, e2, en, j1, j2, c2 (LRM/PRLM), sa, h2	L2, L3, L4 also L4	1 Hz	Global + European regions + Arctic + SW Indian	AVISO+	Widely used reference dataset processed with standard techniques. Distribution of global, Mediterranean Sea, Black Sea products is migrating to CMEMS during 2016
CMEMS	CLS CNES	e1, tx, e2, en, j1, j2, c2 (LRM/PRLM), sa (s3a to be added soon)	L3 L3 for assimilation	1 Hz	Global + European regions	marine.copernicus.eu	Marine environment monitoring service of the EC/ESA Copernicus programme, providing products and services for all marine applications
*PISTACH*	*CLS* *CNES*	*j2*	*L2*	*20* *Hz*	*Global*	*AVISO+*	*Experimental Jason-2 products for hydrology and coastal studies with specific processing. Will be discontinued at the end of 2016 in favour of PEACHI*
*PEACHI*	*CLS* *CNES*	*sa, (j2 to be added soon)*	*L2*	*40* *Hz*	*Global*	*AVISO+*	*Experimental SARAL/AltiKa products including dedicated retracking and corrections leading to more accurate products for coastal zones, hydrology and ice. From 2017 expected to generate also j2 products*
*X-TRACK*	*LEGOS-CTOH*	*tx, j1, j2, gfo, en*	*L2, L3*	*1* *Hz* *20* *Hz* *(test)*	*23 regions*	*CTOH* *AVISO+*	*Specific processing using improved data screening and latest corrections available (see Sect.* 6 *)*
RADS	EUMETSAT, NOAA, TUDelft	gs, e1, tx, pn, e2, gfo, j1, n1, j2, c2, sa	L2	1 Hz	Global	TUDelft	Widely used dataset, mirrored by tens of sites worldwide, with continuously updated corrections, but no specific coastal processing
*ALES*	*NOC*	*j2, n1, (j1, j3 to be added soon)*	*L2*	*20* *Hz*	*Global, <50* *km from coast*	*PODAAC*	*Experimental products from the ALES processor included in SGDR-type files alongside the standard products and corrections*
*SARvatore*	*ESA-ESRIN*	*c2 (SAR only)*	*L2*	*20* *Hz*	*SAR mode regions*	*ESA GPOD*	*On-demand Processing service for the CryoSat-2 SAR mode data where the user can configure some processing parameters to meet specific requirements (for istance for the coastal zone)*
*COP*	*ESA*	*c2 (LRM/PLRM)*	*L2*	*20* *Hz*	*Global*	*ESA*	*Global products for CryoSat-2 from an ocean processor (output is in PLRM over the SAR mode regions)—but no specific coastal processing*

The italic text highlights those products that are provided at a higher post rate (20 or 40 Hz, corresponding to along-track distances of approximately 350 and 175 m, respectively) and are therefore more amenable to coastal altimetry applications

The abbreviations used for the altimeters are gs, Geosat (1985–1989); e1, ERS-1 (1991–1996); tx, TOPEX (1992–2002); pn, Poseidon (1992–2002); e2, ERS-2 (1995–2011); gfo, Geosat Follow-On-1 (2000–2008); j1, Jason-1 (2002–2013); en, Envisat (2002–2012); j2, Jason-2 (2008-present); c2, CryoSat-2 (2010-present); sa, SARAL/AltiKa (2013-present); h2, HY-2A (2014-present); j3, Jason-3 (2016-present); s3a, Sentinel-3A (2016-present). For CryoSat-2 (c2), a further specification is added when data are only available from the low-resolution mode and pseudo-low-resolution mode (LRM/PLRM) or only from the SAR mode regions. The abbreviations used for product levels are L2, along-track data with corrections; L3, data gridded on regular grids in space and time; L4, products derived from analysis of multiple measurements, such as climatologies

### Strategies for Improving the Coastal Altimetry Data

Let us first recall the basic equation by which the fundamental measurement taken by a radar altimeter, i.e. a measurement of range from the instrument to the sea surface, is converted into an accurate measurement of the surface height:

1 $$ Surface\_height = orbital\_altitude{-}\left( {range + corrections} \right) $$

where *orbital_altitude* is the height of the satellite centre of mass with respect to a reference surface (typically a reference ellipsoid) which is normally modelled to an accuracy of 2–3 cm by using a combination of GPS positioning, laser ranging and radio positioning using ground stations. The *Surface_height* so obtained contains the geoid variations as well as the oceanographic signal; subtraction of a mean sea surface^3^ removes the time-invariant geoid and the mean of the dynamic topography and yields the SLA (provided also the tidal signal and the atmospheric signals are corrected for).

In the coastal zone, in addition to the refinement of the statistical techniques for screening and filtering of the various data and corrections (such as in X-TRACK), there are two complementary courses of actions for improving the quality of the retrieved data: (1) applying specialized retracking (i.e. improving the estimation of the *range* term in Eq. 1) and (2) applying improved corrections for the atmospheric, surface or geophysical effects (i.e. improving the *corrections* term).

Reflected radar pulses returning to the altimeter receiver are recorded against time in the so-called waveforms. These are sent to the receiving station on the ground and ‘retracked’, i.e. fitted with a waveform functional form (waveform model) to yield the fundamental measurements of range (from which sea level is measured), SWH and radar backscatter (in turn related to wind). The fitting is usually carried out via least squares or maximum likelihood algorithms. Over the open ocean, waveforms normally conform well to the Brown model (Brown 1977; Hayne 1980). In a band typically extending ~10 km from the coastline, a significant portion of the radar waveforms depart from the Brown model (this portion gets larger approaching the coast, as shown in Fig. 3 of Halimi et al. 2013), calling for modified retracking strategies. The factors that impact on the waveforms are not only the presence of land in the altimetric footprints, but also the occurrence of ‘bright targets’ in the footprint such as patches of very calm water in sheltered areas (Gómez-Enri et al. 2010). A summary of the various strategies proposed in recent years for coastal retracking is in Passaro et al. (2014); these strategies include the use of a modified functional form (as in Halimi et al. 2013), the pre-classification of waveforms and the retracking of ‘sub-waveforms’, i.e. a portion of the waveform unaffected (or less affected) by coastal artefacts (as in Yang et al. 2012).

**Fig. 5:**
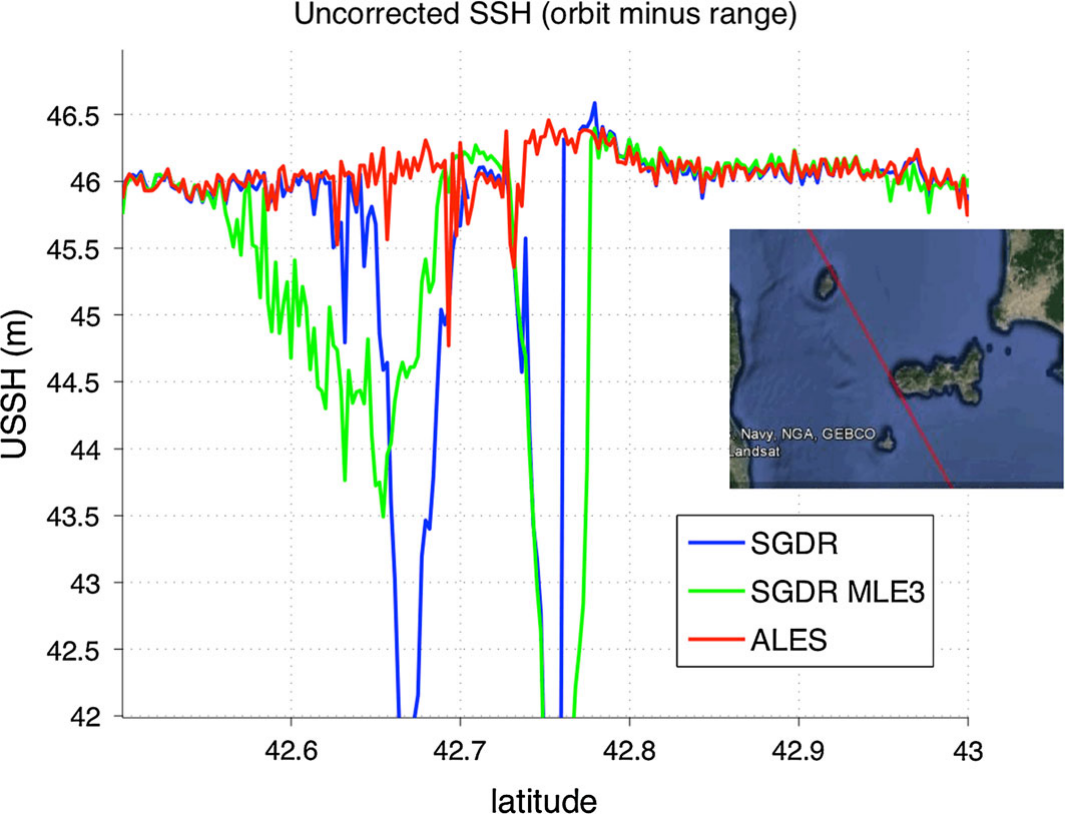
Example of improved retrieval of sea level (here labelled as sea surface height or SSH) close to the coast via a specialized algorithm. The *inset* shows a map of Jason-2 descending pass 0044 (*red* track) in the vicinity of Elba Island in the Mediterranean Sea. The *line* plots show the 20-Hz uncorrected SSH (i.e. orbital altitude of the satellite minus retracked range) measured during the overpass of that ground track during orbital cycle 252 (at 13:40 on 7 May 2015) using three different retrackers: the standard Brown 4-parameter available in the sensor geophysical data records (‘SGDR’, in *blue*), the 3-parameter maximum likelihood estimator also in the SGDR (‘SGDR MLE3’, in *green*), and the ALES retracker (Passaro et al. 2014). The ALES estimates are much less affected by the proximity to the Elba Island coast within 42.65 and 42.8°N

A solution recently proposed to improve the retrieval of sea level, SWH and wind in the coastal zone is the Adaptive Leading-Edge Subwaveform (ALES) algorithm (Passaro et al. 2014). ALES is a two-pass retracking algorithm, based on the Brown model, where the second-pass sub-waveform window is selected based on the first-pass estimates of SWH, in a way that minimizes the performance degradation w.r.t. an ideal, uncorrupted full-waveform case. This algorithm has been validated for sea level and SWH and used for different altimeters in number of case studies (Passaro et al. 2014, 2015a, b, 2016; Gómez-Enri et al. 2016). Figure 5 shows an overpass of Jason-2 tangent to the coast of Elba Island in the Mediterranean Sea, where standard retracking algorithms yield unrealistic metre-level variations of the sea level while the adoption of ALES visually improves the height profile. This kind of qualitative assessment of course needs to be followed up by a quantitative validation of the data, whose scope and methods depend to some extent on the intended application, and some validation has been carried out already in the papers cited above, while further comparison between ALES data and tide gauges around the coast of the UK is presented in Sect. 5.

The improvements in retracking have been accompanied by equally important improvements in some of the corrections that need to be applied to altimetry data to account for atmospheric path delays and other geophysical effects. The two major improvements are in the correction of the path delay due to tropospheric water vapour (‘wet tropospheric’ correction, see Obligis et al. 2011) and in the tide models that are needed for all those applications where the tidal component is not part of the observed signal and need to be removed (Ray et al. 2011). Here we will briefly summarize the main advances in the wet tropospheric correction, while for the improvement in tidal models we refer to the comprehensive review by Stammer et al. (2014).

The wet tropospheric correction is almost proportional to the integrated water vapour content of the atmosphere. Over the open ocean, it is either directly measured by a 2- or 3-channel passive microwave radiometer on board some altimeters, or can be estimated with good accuracy using meteorological models, which however lack spatial structure. On approaching the coastal zone, the radiometer-based measurements degrade rapidly when land enters the radiometer footprint, which has a 10–50 km diameter depending on the particular channel. Models, on the other hand, are still not particularly able to capture the shorter-scale changes in water vapour in the coastal zone and lack accuracy. The need for an improved correction has been apparent since the inception of coastal altimetry and several solutions have been proposed (Obligis et al. 2011). Notable contributions include the improved algorithm proposed by Brown (2010) and applied to the advanced microwave radiometer on the Jason-2 mission, with an estimated error less than 1.2 cm within 5 km from land. Another successful improvement is the GPD correction by Fernandes et al. (2015), built by combining passive microwave measurements from altimetric missions with path delays measured by a network of coastal GNSS stations, and being extended to include measurements from other imaging microwave radiometers. This has been applied globally to 8 missions in the ESA Sea Level CCI project and has yielded a significant impact on regional sea level trends with particular relevance to the coastal and polar regions, due to an efficient correction for land and ice contamination in the radiometer footprint (Fernandes et al. 2015).

**Fig. 6:**
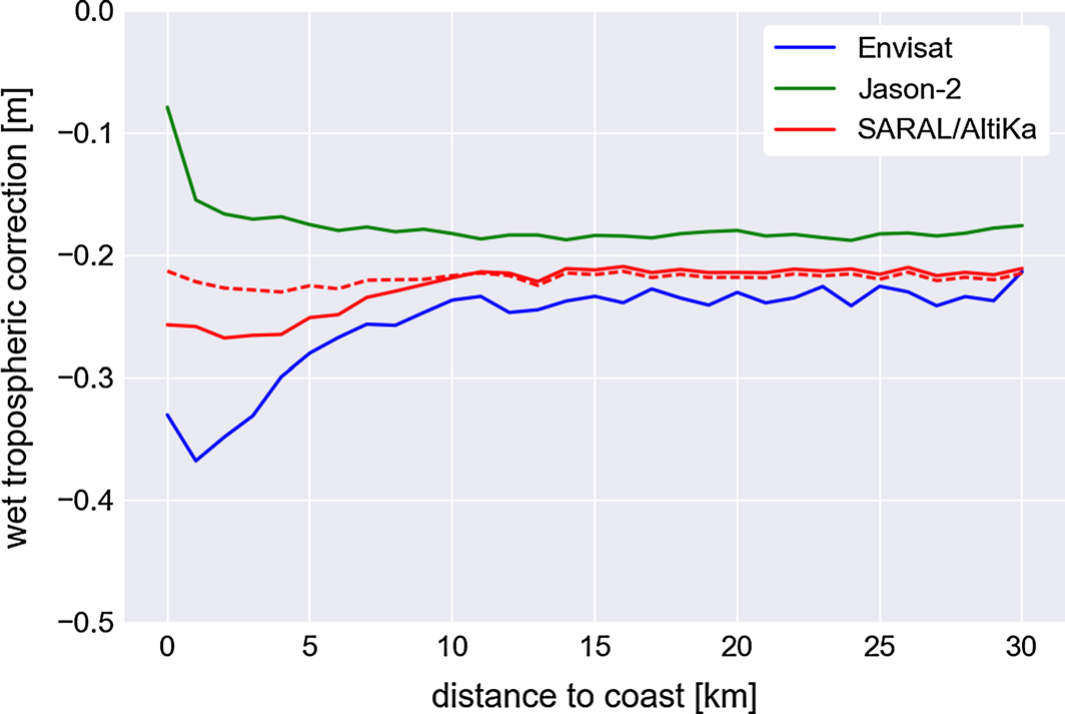
Mean wet tropospheric correction as a function of distance to coast for SARAL/AltiKa, Jason-2 and Envisat data. A specific coastal algorithm is applied on Jason-2 data, and the dotted red line corresponds to an equivalent coastal processing applied on SARAL/AltiKa data

The impact of the wet tropospheric correction in the coastal zone is well illustrated by Fig. 6, which shows the mean value of this correction as a function of distance to coast for Jason-2, Envisat and SARAL/AltiKa data. For Jason-2 the wet tropospheric correction has been computed with an improved algorithm, i.e. Brown (2010), showing a much smaller, more realistic variation in the last few kilometres to the coast than the ‘standard’ corrections derived from the AltiKa and Envisat dual-channel radiometers (red and blue solid curves, respectively), which both start degrading shorewards of 10 km from the coast.

## The Potential of New Altimetric Technologies in the Coastal Zone

The launch of two satellites—CryoSat-2 and AltiKa with two important technological improvements, i.e. SAR mode altimetry and Ka-band altimetry— has opened new prospects for altimetry. In this section, we describe the potential of these two technologies for monitoring sea level in the coastal zone, where those two missions perform particularly well.

### Ka-Band Altimetry: AltiKa

The SARAL/AltiKa French/Indian satellite altimetry mission was launched on 25 February 2013. The platform embarks a DORIS antenna and GPS receivers for precise orbit determination, a dual frequency radiometer for wet tropospheric path delay retrieval, and is the first mission to carry a Ka-band (36.5 GHz) altimeter providing data at the high posting rate of 40 Hz, corresponding to ~180 m along the ground track of the satellite. Compared to previous altimeters that are using the Ku-band at 13.6 GHz (and 20-Hz posting rate), SARAL/AltiKa is expected to provide better vertical resolution of the range thanks to a larger bandwidth, and improved horizontal resolution thanks to a narrower antenna beam (footprint diameter is only 8 km compared to 20 km on Jason-2), at the cost of higher sensitivity to rain events (Vincent et al. 2006; Steunou et al. 2015). The high precision measurements provided by the altimeter are very valuable for the characterization of coastal sea level and dynamics, which is one of the main scientific objectives of the SARAL/AltiKa mission (Verron et al. 2015). The current geophysical data record (GDR) products (GDR-T patch 2 version) are dedicated to open ocean, but the CNES PEACHI prototype (Valladeau et al. 2015) processes high rate data with up-to-date algorithms for different surfaces (coastal, ice, hydrology). These products are available through the ODES portal (http://odes.altimetry.cnes.fr).

**Fig. 7:**
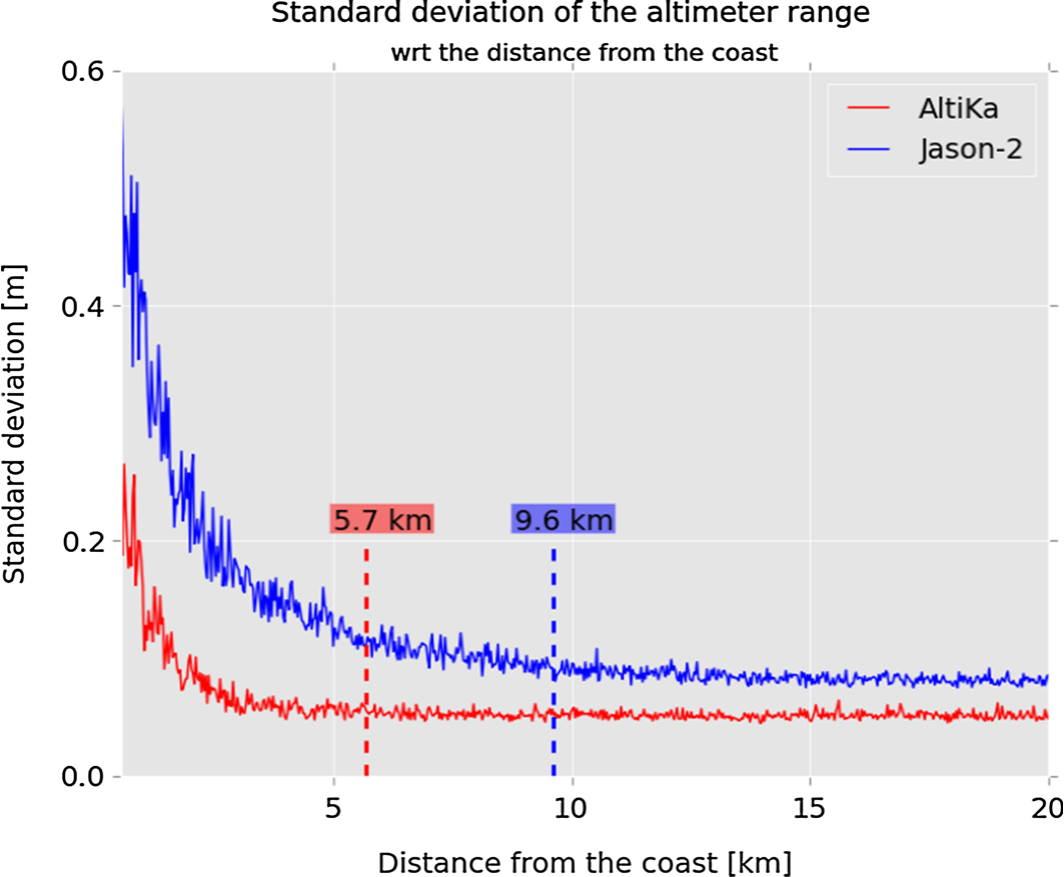
Standard deviation of the altimeter range as a function of the distance to the coast for Jason-2 and SARAL/AltiKa data

Calibration and validation activities demonstrated the excellent performance of SARAL/AltiKa data over the open ocean (Prandi et al. 2015). At the coast, data quality remains very good with little land contamination of the altimeter measurements up to 5 km from the coast. Figure 7 illustrates that by showing the evolution of the standard deviation of the altimeter range with respect to distance from the coast, which is directly related to the instrumental noise level. For Jason-2, the noise level rises at about 10 km from the coast. For SARAL/AltiKa, the noise level remains fairly constant, and at the same level as over the open ocean, down to 5 km from the coast or less. Another issue with coastal measurements is the wet tropospheric path delay retrieval from radiometer brightness temperatures, which can be impacted by land contamination as far as 50 km from the coast. Figure 6 displays the wet tropospheric correction derived from the radiometer measurements as a function of distance to coast for different missions. Envisat data show a drop by about 20 cm from 10 km onwards while Jason-2 remains stable thanks to the dedicated processing applied (Brown 2010). On SARAL, with no dedicated processing applied, the drop is reduced with respect to Envisat (about 10 cm, solid red line), while a simple coastal processing (extrapolation of the last uncontaminated brightness temperature) allows the wet tropospheric correction to remain very stable near the coast (dotted red line).

These altimetry-based assessments of instrumental performances can be completed by comparisons to in situ measurements to demonstrate the coastal capabilities of the SARAL/AltiKa mission. Several studies have used SARAL/AltiKa data in coastal zones, and their results tend to confirm what instrumental quality assessment suggests. Troupin et al. (2015) compared ocean currents derived from SARAL/AltiKa altimetry, HF radar and glider measurements and found a good agreement between altimeter and glider currents, as close as 10 km from the coast. Similar results were also found by Pascual et al. (2015), with further improvements foreseen from dedicated near-coast instrumental algorithms and geophysical corrections. Birol and Niño (2015) compared Jason-2 and SARAL/AltiKa data in coastal areas of the north-west Mediterranean Sea. They got a much better sampling from SARAL/AltiKa (more data available), and comparisons with local tide gauges showed a better agreement than with Jason-2, both for correlation (0.7 vs. 0.54) and RMS error (3.3 vs. 4.2 cm). Hareef Baba Shaed et al. (2015) compared significant wave heights (SWH) from SARAL/AltiKa with wave buoy measurements along the coasts of India and found correlations ranging from 0.85 to 0.98 and RMS errors lower than 0.4 m.

All the above examples demonstrate the capabilities of Ka-band altimetry for the monitoring of coastal ocean dynamics. With a complete reprocessing foreseen in 2017, and with its long-term stability validated with comparison with other altimeters and tide gauges as is being done in the Sea Level CCI project, the 3+ years long SARAL/AltiKa record will provide even more valuable data for coastal studies, providing extended observations of sea level over the same 35-day repeat set of orbits sampled by ERS-1, ERS-2 and Envisat between 1992 and 2010.

### SAR Mode Altimetry: CryoSat-2

ESA’s CryoSat-2 satellite was launched on 8 April 2010, with the primary mission role of monitoring the cryosphere by measuring variations in ice thickness, but has been proven of exceptional utility also for the monitoring of the oceans (see for instance Dibarboure et al. 2012). A technological innovation of CryoSat-2 Synthetic Aperture Interferometric Radar Altimeter (SIRAL) is the delay-Doppler mode of measurement (Raney 1998) which we will refer to as ‘SAR mode’ as it involves an unfocused along-track synthetic aperture radar (SAR) processing of the radar echoes. When in SAR mode, SIRAL exploits the Doppler information in the returned pulse to achieve a much finer resolution in the along-track direction (the width of the along-track SAR resolution cell is ~350 m), virtually independent of the sea state. The size of the altimeter footprint in the across-track direction is the same of a conventional altimeter, i.e. 2–20 km depending on the sea state. By averaging independent measurement from adjacent cells, the augmented along-track resolution can be traded off, all or in part, to achieve lower noise on the estimated parameters. Due to power and data downlink constraints, SIRAL on CryoSat-2 can only be operated in SAR mode (and in another experimental mode, SAR interferometry, of primary use over ice surfaces) over a small portion of the Earth’s surface, and is instead in conventional low-resolution mode over most of the surface. In practice, this means that SAR mode data are available over a number of ‘patches’, one of which covers the entire European coastal sector. The importance of CryoSat-2 is magnified by the fact that SIRAL is a precursor of the Synthetic aperture Radar ALtimeter (SRAL) on the Copernicus Sentinel-3 satellites due to provide systematic oceanographic observations for the next 20 years (Donlon et al. 2012). Sentinel-3A was launched on 16 February 2016 and is being operated in SAR mode over the entire global ocean.

**Fig. 8:**
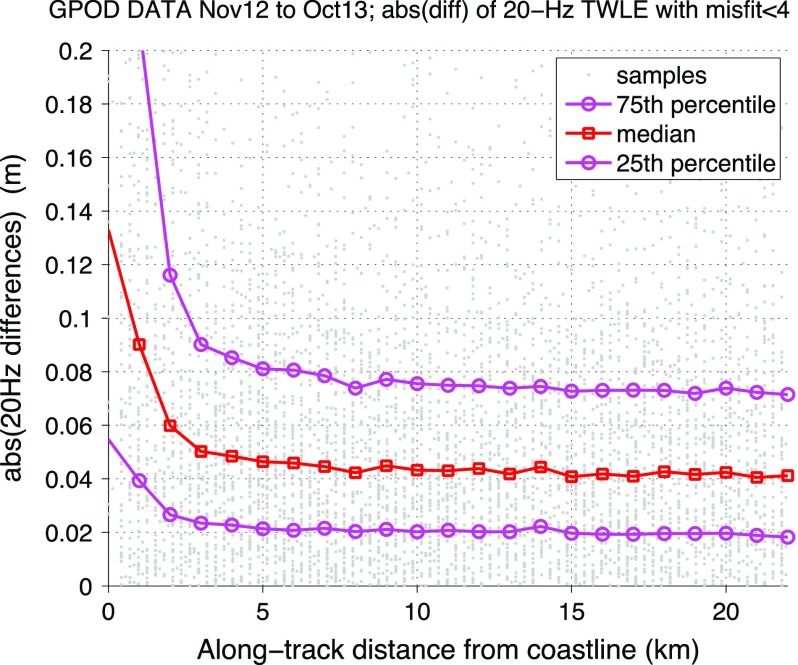
Example of coastal performance of SAR altimetry. Scatterplot of noise values (estimated as the absolute value difference between consecutive Total Water Level Envelope (TWLE) measurements) against along-track distance from coast, and the statistics of its distribution in 1-km distance bins, for CryoSat data around the coast of the British isles reprocessed with the GPOD SARvatore processor (Dinardo 2014) within the ESA CP4O project (Cotton et al. 2015). The data have been screened based on a threshold on retracking misfit. From Cipollini and Calafat (2016)

The performance of SAR mode altimetry in the coastal zone has been recently assessed using CryoSat-2 data around the coast of the British Isles within the ESA CP4O project (Cotton et al. 2015; Cipollini and Calafat 2016). The noise level in the altimeter measurements has been computed as the absolute value difference between consecutive values (at 20-Hz posting rate) of the total water level envelope or TWLE (i.e. the SLA inclusive of tides and atmospheric forcing; the results would be virtually the same by using SLA), and its variation in the coastal strip has been studied. Figure 8 shows a representative result of this analysis, with a scatterplot of noise and its statistics in 1-km bins of distance from coast. The median of the distribution in particular is a good indicator of the ‘typical’ level of noise, and it can be seen that this median stays flat at 5 cm or less from the open ocean up to 3 km from the coast and it is still relatively low (6 cm) at 2 km and 9 cm at 1 km.

Results such those presented are extremely encouraging in terms of demonstrating the low noise level in SAR altimetry data due to the excellent performance of the radar, but for the particular application to long-term monitoring of sea level what is paramount is the stability of the whole measurement system, including the corrections. As for AltiKa, this is currently being investigated in the ESA Sea Level CCI project.

## A Case Study Around the Coast of the UK

As an example of how the local sea level can be monitored with altimetry and tide gauges, which also allows investigating the link between deep-ocean and coastal sea level variability, we present here the results of an analysis of sea level around the UK coastline, which was conducted within the framework of the Sea Level SpaceWatch project. This project was funded by the UK Space Agency within the Space for Smarter Government Programme to design and prototype an operational service delivering systematically updated sea level observations around the UK, from a combination of satellite altimeter observations and tide gauge measurements (Cotton 2016). The focus of the analysis presented here is on the annual cycle of sea level over the period 2002–2015, but first we also present a comparison between the altimetry and tide gauge observations on interannual timescales, as a form of validation.

Here we use along-track altimetry data from Jason-1 and Jason-2 as reprocessed by the coastally adapted ALES retracker (Passaro et al. 2014) described in Sect. 3.1 and covering the period 2002–2015. We use a total of 58 tide gauge records, of which 46 were obtained from the data archives of the British Oceanographic Data Centre (BODC), 11 from the UK Coastal Channel Observatory (CCO), and 1 from the Port of London Authority (PLA). The temporal resolution of the tide gauge data is 15 min for records stored at the BODC and 10 min for those stored at the CCO and PLA. For consistency with the satellite altimetry data, the atmospheric correction was applied to the tide gauge data. In particular, we used the dynamic atmospheric correction (DAC) provided by AVISO (ftp.aviso.altimetry.fr), which consists of the barotropic response of the ocean to wind forcing and atmospheric pressure as estimated by the Mog2D-G model for periods shorter than 20 days and the inverse barometer (IB) approximation for longer periods. The DAC data are provided in the form of 6-h sea level fields on a 1/4° × 1/4° regular grid covering the global oceans. The atmospheric correction at each tide gauge is taken from the nearest DAC grid point to the tide gauge.

The amplitude, *A*, and phase, *ϕ*, of the annual cycle are estimated by first fitting the following linear model to the sea level data:

$$ y_{t} = a + bt + c\cos \left( {\frac{2\pi }{365.24}t} \right) + d\sin \left( {\frac{2\pi }{365.24}t} \right) + e_{t} $$

where *e*_*t*_ is the error term. And then, once the regression coefficients have been estimated, by

$$ A = \sqrt {c^{2} + d^{2} } $$

$$ \phi = \arctan \left( {d/c} \right) $$

As an initial validation test, we compare de-seasoned and de-trended time series of sea level from satellite altimetry and tide gauges at each station. Because altimetry measurements are not, in general, taken at the exact location of a tide gauge, but at some point nearby, nor are they collocated in time with the tide gauge observations, some processing is necessary to obtain consistent altimeter–tide gauge pairs for the comparison. It is clear that altimetry measurements taken too far from a tide gauge station may not be relevant to the sea level measured by the tide gauge and thus a search radius centred on each tide gauge needs to be imposed. The approach taken here is to test for different values of the radius within the range 0–200 km and find that minimizing the root-mean-square difference (RMSD) between the tide gauge and altimetry observations. The advantage of this approach is that the selected radius represents the optimum radius for each tide gauge individually and thus different radiuses are used for different tide gauges. Then for each altimetry pass, an altimetry value is obtained by computing the median (which is more robust against outliers) of all records falling within the selected radius. The corresponding tide gauge matching value is obtained by linearly interpolating the tide gauge observations to the time of the altimetry pass. The resulting time series were then converted to monthly values of sea level by averaging all available values within each month.

**Fig. 9:**
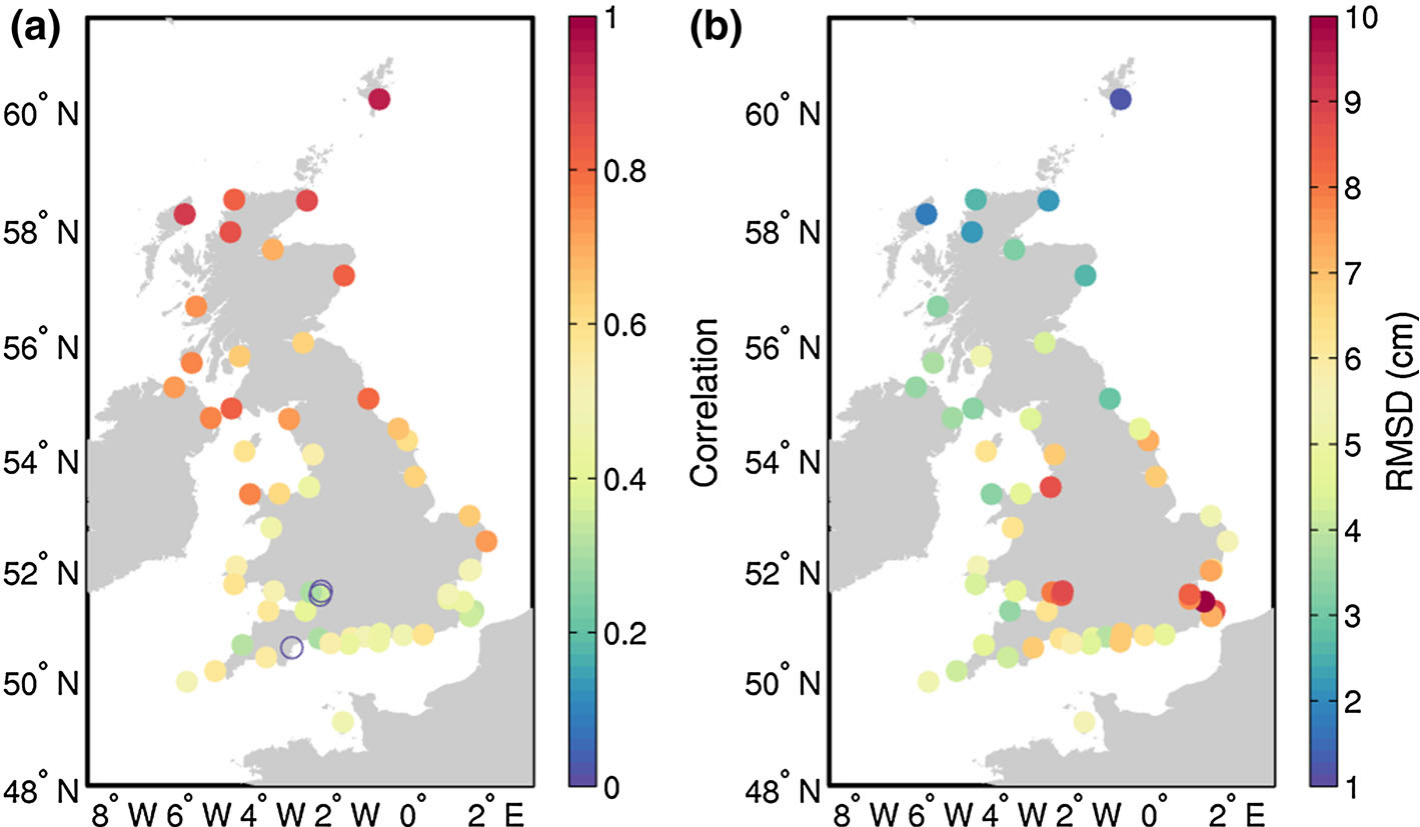
Correlation (**a**) and root-mean-square difference (RMSD) (**b**) between de-seasoned and de-trended sea level from altimetry and tide gauge observations. *Empty circles* in **a** denote non-significant correlation

The correlation between the altimetry and tide gauge time series is significant at all 58 stations except Portbury, Severn Bridge and Teignmouth (Fig. 9a). Higher correlations are found at stations in the Irish Sea, the North Channel, the Atlantic Ocean and the North Sea, with an average value of about 0.75, whereas lower correlations are found at stations in the English Channel with an average value of about 0.45. Regarding the RMSD (Fig. 9b), there is a clearly defined spatial pattern consistent with that found for the correlation, with the northern stations showing lower RMSD values (~3.8 cm) and stations in the English channel showing larger differences (~5.8 cm). Residual tidal errors (after the correction) may play a role in those differences, but the issue warrants further investigation.

**Fig. 10:**
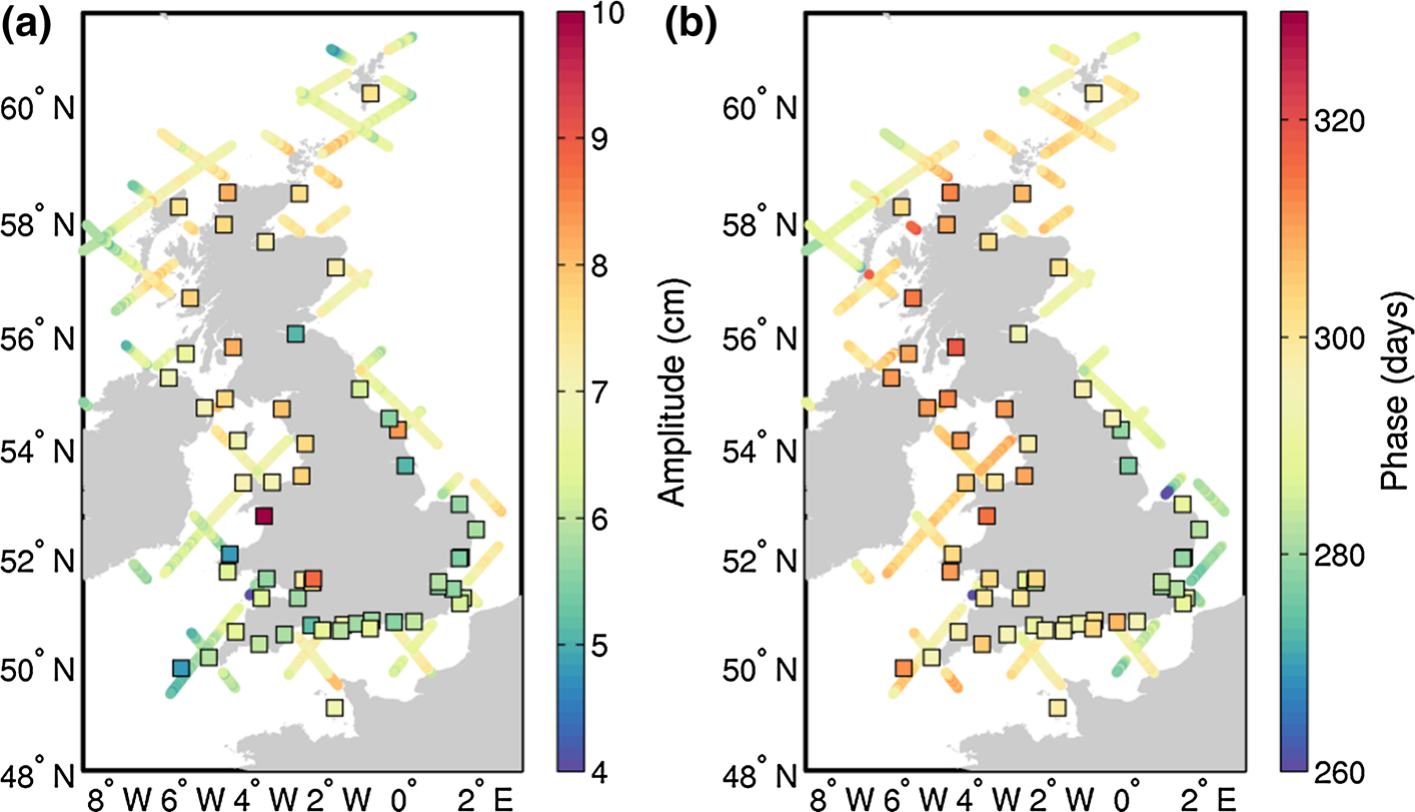
Annual amplitude (**a**) and phase (**b**) of the mean annual cycle from along-track satellite altimetry measurements and from tide gauge observations (*squares*)

There is also a good agreement between satellite altimetry and tide gauge observations in terms of the annual cycle, for both its amplitude (Fig. 10a) and phase (Fig. 10b). The amplitude of the annual cycle ranges from 5 to 9 cm at all station with the exception of the Barmouth tide gauge, which due to its location seems to be affected by river Mawddach and shows a very large annual amplitude of 85 cm. From Fig. 10a, we note that the amplitude of the annual cycle tends to be larger in the northern coasts of the Great Britain and smaller in the English Channel. Regarding the phase of the annual cycle (Fig. 10b), we see that the cycle peaks in early October along the south-east coast and over a month later in mid-November along most of the west coast of Great Britain. Such geographical non-uniformity in the phase of the annual cycle is observed both in the tide gauge observations and the altimetry data.

## Evolution of Sea Level Trend from the Open to the Coastal Ocean

As seen in the previous sections, altimetry already provides unique observational datasets to characterize how sea level variability evolves from the open ocean to the coastal ocean, and new technologies promise further improvements. An obvious scope arises to characterize the statistics of long-term sea level variations as functions of the distance from the coast, which would help to establish a quantitative link between open-ocean and coastal sea level variations at temporal scales relevant to climate. One particularly intriguing aspect is the coastward evolution of sea level trends. No conclusive causes supporting a difference of the coastal trend from the open ocean one have been identified so far when those trends are averaged over the entire globe (see Scharroo et al. 2009). So, the global mean sea level rise at the coast is expected to coincide with the rate from open-ocean altimetry like that from the Sea Level CCI in Fig. 3, but this agreement will have to be confirmed by coastal altimetry. However, differences are certainly possible at a regional level, due to changes in strength and location of coastal currents and changes in the coastal winds. For those reasons, the issue of the variation of sea level with distance from the coast remains worth investigating. To provide some insights into this issue, we present in this section a case study over a region off the western coast of Africa. This region has been chosen both because it is particularly vulnerable to sea level rise (Cazenave and LeCozannet 2014), and because it has a relatively simple coastline, so it is close to an idealized situation. We use along-track nadir altimetry data from the TOPEX/Poseidon, Jason-1 and Jason-2 satellites, which share the same ground tracks and provide the longest sea level anomaly time series, over the 1993–2012 period. Three datasets are analysed and compared:

- the standard AVISO dataset (version 2014 of delayed-time data,) distributed by the Copernicus Marine Environment Monitoring Service (CMEMS). These are the ‘vfec’ data, i.e. validated, filtered, sub-sampled and corrected for long wavelength errors; the spatial sub-sampling of data results in a spatial resolution of 14 km.
- two datasets provided by LEGOS/CTOH, in which along-track altimetric data have been reprocessed using the 2011 and 2016 versions of the X-TRACK algorithms, respectively (Birol et al. 2016). These are more adapted to coastal regions and provide data at a spatial resolution of 7 km. The editing and extrapolation of geophysical corrections has been improved in X-TRACK2016 compared to X-TRACK2011, on two aspects: (1) the removal of land contamination in the radiometer-derived wet tropospheric correction is now performed in X-TRACK2016 with an algorithm computing the proportion *p* of land within the radiometer footprint of each altimeter mission and rejecting the wet tropospheric correction values for which *p* > 0. Possible other outliers away from the coast are discarded by an algorithm that detects large differences between the correction values at two consecutive points along the track for each cycle. The resulting data gaps are either interpolated (away from the coast) or filled with the closest values qualified as valid (in the land/sea transition areas). A technique based on the discrete wavelet transform is then used to compute a cleaned and noise-free wet tropospheric correction; (2) the ionospheric correction filtering has been updated to more efficiently detect outliers: the filter is a median absolute deviation (MAD) threshold in X-TRACK2016, used instead of the 3*σ*-threshold filter used in X-TRACK 2011 (*σ* being the standard deviation). The sea-state bias (SSB) correction was smoothed in X-TRACK2011 using Bezier curves. In X-TRACK2016, the SSB correction is filtered in the along-track direction with a Loess low-pass filter, and missing values are replaced by the nearest interpolated data. Regarding the other altimeter corrections, usually derived from models, very few values are discarded by the editing process. We choose to replace flagged corrections by their nearest valid neighbours.

Along-track data from the three datasets were post-processed with various filters to remove the remaining erroneous data, using a strategy similar to that used in Melet et al. (2010). As the coastal shelf is narrow off Western Africa (a few tens of km), we selected the sections of the altimetry tracks that are located less than 200 km off the coast (referred to as coastal sections hereafter) to characterize the evolution of sea level trends from the open to the coastal ocean. For each coastal section, the changes in sea level trend were calculated relative to the ‘open ocean trend’ for that section (defined as the mean trend for the part of the section located in the open ocean from 160 to 200 km off the coast; this choice was made to exclude the continental shelf).

**Fig. 11:**
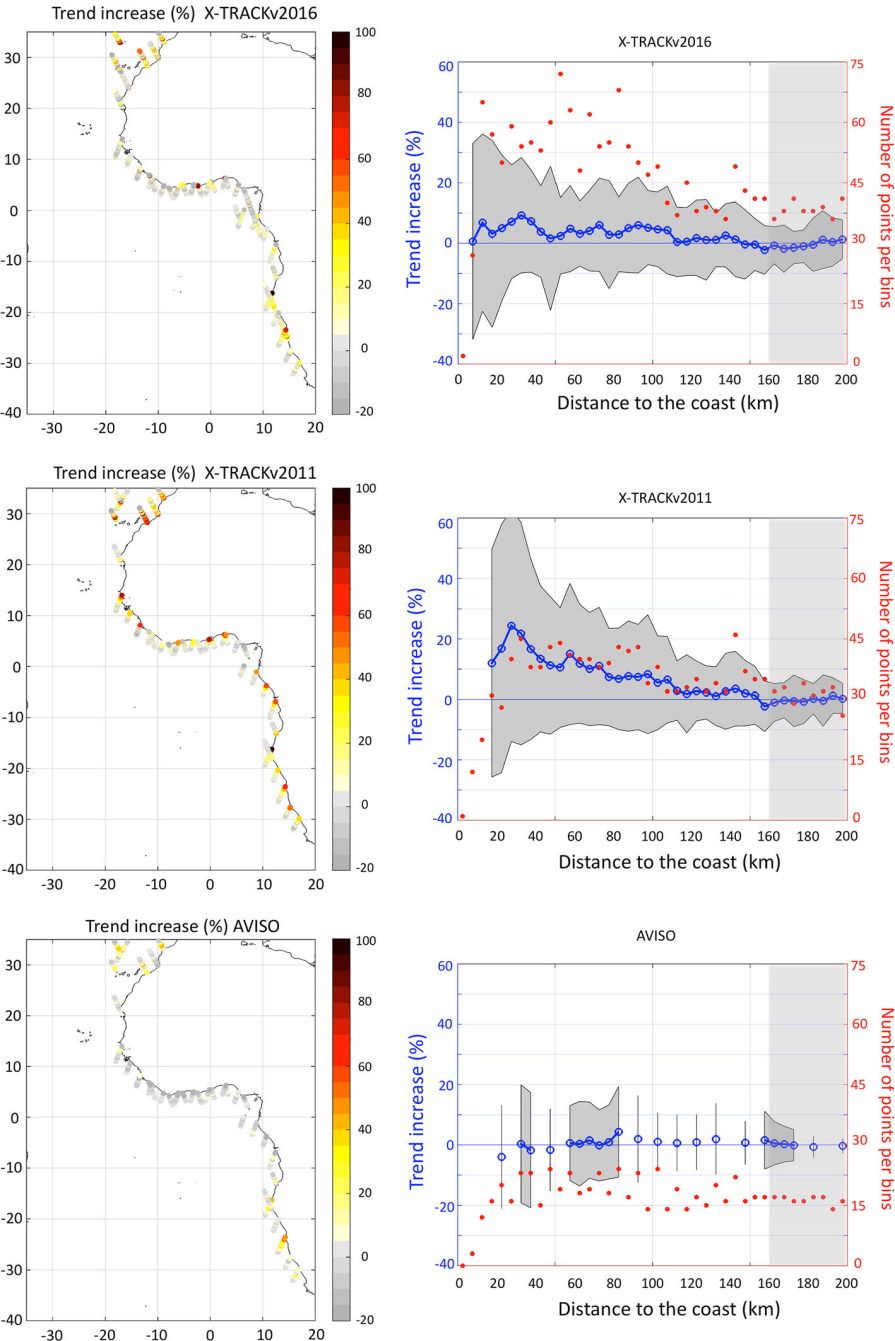
(*Left*): Relative changes in SLA trend (mm/year) over 1993–2012 in percentages along coastal sections of altimetry tracks offshore Western Africa for the (*top*) X-TRACKv2016, (*middle*) X-TRACKv2011 and (*bottom*) AVISO along-track datasets. Only the sections of altimetry tracks located less than 200 km off the African coast are studied. Changes are relative to the open-ocean trend defined here as the trend of sea level anomalies averaged over the sections of altimetry tracks located from 160 to 200 km offshore the African coast. (*Right*): Relative changes in SLA trend (in %) were averaged as a function of the distance to the coast, using 5 km wide bins (*blue line*, *left axis*) for the (*top*) X-TRACKv2016, (*middle*) X-TRACKv2011 and (*bottom*) AVISO along-track datasets. The *grey* envelope shows plus and minus one standard deviation from the average. The number of valid points used to compute the mean and standard deviation of SLA trend changes for each bin is shown in *red* (*right axis*). As in the *left panels*, changes are relative to the trend over the 160–200 km off the coast band (this reference part is shown in *light grey shading*). Results are only shown for bins in which at least 15 coastal sections had valid points for this bin for the calculation of the mean and standard deviation of the trend. Results are based on the TOPEX/Poseidon, Jason-1 and Jason-2 missions over 1993–2012

The number of valid independent measurements for which the sea level trend was computed in the coastal sections is larger in the X-TRACKv2016 than in the X-TRACKv2011, especially in the last few kilometres towards the coast, due to a better editing of the data and to a more refined processing of geophysical corrections applied to altimetric data in X-TRACKv2016 than in X-TRACKv2011 (Birol et al. 2016). The number of measurements is much lower in the AVISO dataset (Fig. 11, right panels, in red) and decreases significantly in the last 30 km off the coast due to both the spatial sub-sampling of AVISO data, less adapted geophysical corrections in the coastal zone, and a more conservative editing of the data in AVISO than in X-TRACK datasets.

The changes in sea level trend from the open to the coastal ocean are shown in terms of percentage in Fig. 11 (left panels). In the AVISO dataset, the trend changes from the open to the coastal ocean mostly range from −20 to +20% for the different coastal sections, with a coastward decrease of the sea level trend equatorward of 10°S/10°N, and a coastward increase of the sea level trend poleward of 25°N/25°S. When sea level trend changes are bin-averaged across all the coastal sections as a function of the distance to the coast (Fig. 11, bottom-right panel), no robust evolution of the sea level trend from the open to the coastal ocean can be seen off Western Africa in the AVISO dataset.

In the X-TRACKv2011 dataset, a substantial increase in the sea level trend can be seen for most coastal sections (Fig. 11, middle-left panel). As more valid data are available in the X-TRACKv2011 dataset than in the AVISO dataset, the averaged coastward evolution of sea level trend can be studied closer to the coast (up to 15 km off the coast). On average across the coastal sections, the sea level trend over 1993–2012 steadily increases from the open to the coastal ocean off Western Africa in this dataset, reaching an increase of 25% 25–30 km off the coast (Fig. 11, middle-right panel). The increase weakens in the last few kilometres off the coast and is less robust across the different coastal sections (grey envelope in Fig. 11, middle-right panel).

Results from the X-TRACKv2016 dataset are in between these from the AVISO and X-TRACKv2011 datasets. The sea level trend increases coastward for coastal sections located south of 15°S and north of 25°N (a result qualitatively robust for the 3 datasets), but less so than in the X-TRACKv2011 dataset. Equatorward of 10°N/10°S, no robust coastward evolution of the sea level trend is seen across the different coastal sections. The greater number of valid data in the X-TRACKv2016 dataset allows quantifying the mean coastward evolution of sea level trend up to 5 km of the coast. On average, the sea level trend only slightly increases coastward in the X-TRACKv2016 dataset (by less than 10%), but this is not robust across the coastal sections (Fig. 11, upper-right panel, grey envelope).

These results show that efforts made to improve satellite nadir altimetry products in the coastal ocean allow recovering more data and obtaining more coherent long-term signals in the coastal zone. In particular, the editing and extrapolation of geophysical corrections have been updated and improved in X-TRACKv2016 compared to X-TRACKv2011. Yet, better geophysical corrections themselves are needed to improve the accuracy and reliability of altimetry data in the coastal zone. The analysis performed here highlights that efforts are still needed on the processing of data and on the geophysical corrections applied to satellite data for studying the sea level trend in the coastal zone over the last two decades more robustly.

It should also be noted that the contribution from waves to sea level variability and trend is not considered here as it is removed from satellite altimetry data since the primary focus of satellite altimetry is to study ocean circulation and dynamics. Yet, wave-induced set-up and run-up can contribute to the sea level trend at the coast (Melet et al. 2016).

## Summary and Conclusions

In this contribution, we have reviewed the status of the measurements of sea level in the coastal zone. First we have summarized the centennial-scale observations that we get from tide gauges, and then we have aimed at showing how improved satellite nadir altimetry products in the coastal ocean are now complementing those observations by allowing meaningful measurements and the retrieval of coherent long-term signals in the coastal zone. The trend towards better altimetric data at the coast comes not only from improved processing and corrections, but also because of technological innovations such as Ka-band altimetry and SAR altimetry, and we have discussed the main advantages deriving from those two innovations that can be now appreciated thanks to the AltiKa altimeter on SARAL and the SIRAL altimeter on CryoSat-2 (in turn a precursor of the Sentinel-3 altimeter, which has recently been launched and is being operated in SAR mode over the entire ocean).

We have then illustrated the use of altimetry for coastal sea level studies with two examples. First, in a case study conducted along the UK coast, we have found a very good agreement between coastal altimetry and tide gauge observations, with RMSDs as low as 4 cm at many stations. This has given us confidence to use the combination of altimetry and tide gauges to characterize the annual cycle of sea level along the UK coasts. We found amplitudes ranging from 5 to 9 cm, with larger amplitudes found in the northern coasts of the Great Britain, and peaks between early October in the south-east coast and mid-November in most of the west coast. Then, we have examined the evolution of sea level trend from the open to the coastal ocean along the western coast of Africa, comparing standard and coastally improved products. We observed that different products give different answers regarding the coastward evolution of the sea level trend, and we cannot yet robustly deduce the quantitative evolution of sea level trend from the open to the coastal ocean.

The clear recommendation stemming from what we have presented is that further efforts are still needed to study sea level trends in the coastal zone from past and present satellite missions. Further improvements are expected from more refined processing and screening of altimetric data, but in particular from the constant improvements in the geophysical corrections applied to them, such as wet tropospheric, tides and dynamical atmospheric corrections, which all become noisier when coming near shore. It is worth noting that such improvements in corrections should enable the full coastal exploitation of the data now flowing in from Ka- and SAR altimetry, and in particular the global SAR altimetry data now coming from Sentinel-3. This growing coastal altimetry field is going to support the monitoring of sea level in the coastal zone as well as other complementary applications such as the study of extreme events (storm surges—see for example Fenoglio-Marc et al. 2015) and the validation of coastal wave models.

Finally, it is important to remark that the advances in coastal altimetry detailed in this paper prepare the modelling community for the flux of higher resolution data—not only those now starting to flow in from Sentinel-3A (and that will be continued by Sentinel-3B/C/D due for launch over the next 5 years, and then by the two satellites of the Sentinel-6 mission), but also the wide-swath high-resolution observations expected from the surface water and ocean topography (SWOT) mission, due for launch in 2021. The advent of SWOT should hopefully complete the process of ‘closing the gap’ between altimetric observations and tide gauge observations of sea level and hopefully confirm the full consistency of those two sets of measurements.

## Footnotes

1 The TOPEX/Jason-1/2/3 series of satellite altimeters is on a 9.92-day repeat orbit and has continuous measurements since September 1992. The ERS-1/ERS-2/Envisat/AltiKa series is on a 35-day repeat orbit and has measurements since April 1992 with some gaps, the longest of which is between Envisat’s change of orbit in October 2010 and the start of the AltiKa data in March 2013. From July 2016, AltiKa has definitely left the 35-day orbit, and it is in a drifting orbit phase with no more orbit keeping manoeuvres, so the time series on that orbit have ended.

2 A further extension backwards of the altimetric data record amenable to long-term sea level research could in principle be achieved with ad hoc reprocessing of the GEOSAT mission (1985–1989), and is highly recommended. This will, however, require careful intercalibration with the post-1991 missions, which can be achieved by using carefully selected tide gauges as calibration transfer standards.

3 A mean sea surface (MSS) is the level of the sea due to all those contributions that can be assumed constant in time and can be computed as the temporal mean of sufficiently long time series. More accurate MSS models (for instance DTU15) are built from combinations of multiple altimetric and gravimetric missions. The choice of a specific MSS over another is critical in the coastal zone, and there may be biases between datasets if they are referred to different MSS.
